# Diet Quality and Satisfaction with Life, Family Life, and Food-Related Life across Families: A Cross-Sectional Pilot Study with Mother-Father-Adolescent Triads

**DOI:** 10.3390/ijerph14111313

**Published:** 2017-10-29

**Authors:** Berta Schnettler, Germán Lobos, Edgardo Miranda-Zapata, Marianela Denegri, Gastón Ares, Clementina Hueche

**Affiliations:** 1 Facultad de Ciencias Agropecuarias y Forestales, Universidad de La Frontera, Temuco 4811230, Chile; 2 Centro de Excelencia en Psicología Económica y del Consumo, Núcleo Científico y Tecnológico en Ciencias Sociales, Universidad de La Frontera, Temuco 4811230, Chile; clementina.hueche@ufrontera.cl; 3 Facultad de Economía y Negocios, Universidad de Talca, Talca 3460000, Chile; globos@utalca.cl; 4 LICSA, Núcleo Científico y Tecnológico en Ciencias Sociales, Universidad de La Frontera, Temuco 4811230, Chile; edgardo.miranda@ufrontera.cl; 5 Facultad de Educación, Ciencias Sociales y Humanidades, Universidad de La Frontera, Temuco 4811230, Chile; marianela.denegri@ufrontera.cl; 6 Instituto Polo Tecnológico de Pando, Facultad de Química, Universidad de la República, Pando 4225, Uruguay; gares@fq.edu.uy

**Keywords:** two-parent families, adolescents, healthy eating

## Abstract

Family is a major determinant of children’s and adolescents’ eating behavior. The objectives of the present study were to assess diet quality, eating habits, satisfaction with life, family life, and food-related life in mother–father–adolescent triads, and to identify profiles of families according to family members’ diet quality. Questionnaires were administered to a sample of 300 two-parent families with one child over the age of 10 in the city of Temuco (Chile), including the Adapted Healthy Eating Index (AHEI), Satisfaction with Life Scale (SWLS), Satisfaction with Food-related Life (SWFoL) scale, Satisfaction with Family Life (SWFaL) scales, and questions relating to their eating habits. Positive relationships were found between the diet quality of the family members, particularly between mothers and adolescents. Three family profiles with different diet qualities were identified: “families with an unhealthy diet” (39.3%), “families in which mothers and adolescents have healthy diets, but the fathers’ diets require changes” (14.3%), and “families that require changes in their diet” (46.4%). These findings stress the key role of mothers in determining family diet quality and suggest a positive relationship between diet quality and satisfaction with life.

## 1. Introduction

Overweight and obesity have increased worldwide at an alarming rate in recent decades, affecting people irrespective of age or socioeconomic status [1]. Although obesity prevalence seems to have reached a plateau in several high-income countries, it continues to grow in Latin American countries, which are undergoing rapid nutritional transitions [2]. In the specific case of Chile, the prevalence of adult obesity was 23.2% in 2015 [3], whereas 34.3% of adolescents have been reported to be overweight (25.1%) or obese (9.2%) [4]. Although several factors are involved in obesity, changes in eating behavior caused by the global nutrition transition have been recognized as major determinants of the obesity epidemic [5]. This transition is characterized by decreased consumption of fresh fruit and vegetables, and increased consumption of processed products with high sugar, fat, and salt content [6]. For this reason, interventions aimed at shifting eating behavior have been identified as top priorities for reducing the burden of obesity and non-communicable diseases [7].

In particular, adolescent eating behaviors are influenced by several interrelated factors, and family has been reported to be one of the main determinants [8,9,10,11]. Parents can shape their children’s eating behavior by providing (un)healthful foods at home [9,12,13,14], modeling food choices [8], encouraging healthy eating [9], and promoting family meals [9,13]. Outcomes related to parental modeling can be both positive and negative, depending on behaviors modeled by the parents and behaviors copied by their children [15]. At the same time, although mothers and fathers influence their children’s eating behavior [16], parental influence has been reported to vary widely [17,18]. Hebestreit et al. [13] found that mothers exert a positive influence on their children’s food consumption because they are more likely to adhere to dietary guidelines, whereas fathers primarily influence their children’s intake of unhealthy foods. Similar findings have been reported by Tabbakc and Freeland-Graves [19]. In addition, mothers not only influence their children’s food consumption, but also that of their husbands [18].

The relationship between food and family plays an important role in the prevention and treatment of adolescent obesity [20,21]. There is evidence of a positive association between frequent family meals and healthy diets in adolescents [9,13,22,23], as well as higher diet quality scores based on the Healthy Eating Index (HEI) in adolescents [24] and adults [25]. Higher HEI scores have been associated with a greater frequency of cooking and eating at home, and negatively associated with a higher frequency of eating out [25]. Nevertheless, when healthful parenting practices are low, family meals do not seem to have a positive association with healthy food intake [9].

The affective dimension of meals as a moment of family unity is an important component of the role of food within families [21,26,27]. Family meals prove to be an important ritual for interaction, preserving relationship closeness, resolving conflicts, expressing affection, providing emotional support, and nurturing harmony and function [10,26,27]. All of these components have been associated with healthier eating as well as a lower likelihood of obesity or being overweight among adolescents. In addition, family meals are also important for the well-being of adolescents [22,28].

Subjective well-being is a multidimensional category of phenomena involving emotional responses, positive and negative affect, and global judgments of life satisfaction in different domains [29]. Satisfaction with food-related life is defined as a person’s overall assessment regarding their food and eating habits [30]. Recent studies suggest that satisfaction in the food domain is positively correlated with overall life satisfaction, both in adults and in emerging adults [30,31,32,33,34,35]. In addition, higher levels of life satisfaction and satisfaction with food-related life have been positively associated with better eating habits [32,33], lower prevalence of being overweight or obese [32,33], and greater frequency and importance assigned to family meals [32,33,34,35].

To the best of our knowledge, no studies have assessed the relationship between these variables while studying different family members simultaneously. In parallel, existing research on food parenting practices and family meals has predominantly focused on mothers [17,36,37]. However, fathers also play an important role in their children’s eating habits due to women’s rising participation in the formal labor market. Although the engagement level of fathers in childrearing has increased over the decades [18,37], existing research on diet quality with samples of mother–father–children triads is still scarce [17,38] and limited to developed countries [38].

Although the relationship between mothers and their children’s dietary intake has been extensively studied in the literature [17,37,38], few studies have examined mother–father intakes. Furthermore, even fewer studies have analyzed diet quality in family member triads [17,38]. In particular, no research has been published examining triads in developing countries and dealing with adolescents over 12 years of age [38]. In this context, the objectives of the present study were: (i) to assess diet quality, eating habits, satisfaction with life, family life and food-related life in mother–father–adolescent triads and (ii) to identify family profiles according to family member diet quality.

## 2. Materials and Methods

### 2.1. Participants

This study used a cross-sectional design. Non-probability sampling was used to recruit a sample of 300 two-parent families with at least one adolescent child between 10 and 17 years of age in Temuco, Chile. Participants were recruited from seven schools that serve socioeconomically diverse populations. Directors in each school signed authorization letters to conduct the research with their students and provided a list containing 5145 telephone numbers of parents of students from fifth grade and up (corresponding to a minimum age of 10 years old).

**Figure 1:**
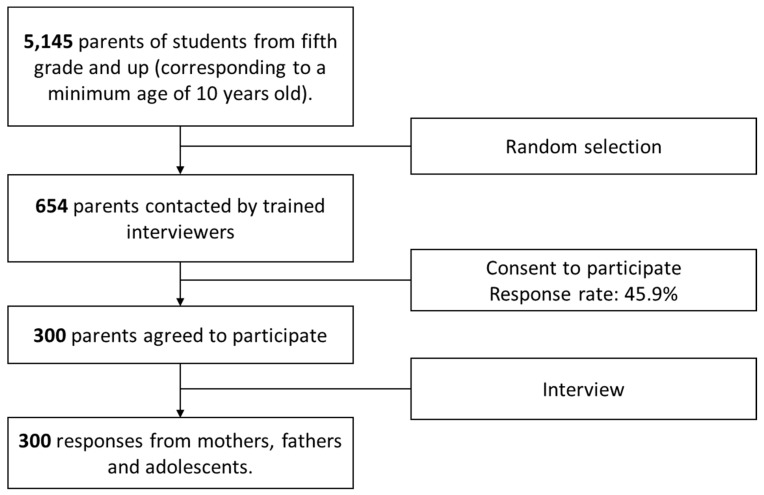
Flow chart of participant recruitment.

From the list of 5145 parents, 654 were contacted by trained interviewers, who explained the study objectives and the strict confidential treatment of the obtained information. Then, the interviewers provided detailed information about the questionnaires and asked if both parents and one of their children between 10 and 17 years of age wanted to participate in the study. A total of 300 parents agreed to participate in the study, resulting in a response rate of 45.9%. Over 50% of the parents who decided to participate in the study had more than one child between 10 and 17 years of age; therefore, the child whose parental contact information was provided by the school was chosen to be interviewed (See flow chart in Figure 1).

Interviews were conducted in participants’ homes or schools, according to their preference. After all family members signed written informed consent forms, the questionnaires were administered by a trained interviewer to both parents and one child over the age of 10. Each family member was interviewed individually without the rest of the family members present. The interviewers read the questions aloud to each family member and recorded the participant responses on paper questionnaires. Respondent anonymity was ensured. The study was conducted between June and December 2016, and the study design was approved by the Ethics Committee of Universidad de La Frontera (Protocol number 005/2016, Chile).

### 2.2. Questionnaire

The questionnaire included the following instruments, which were answered by all family members (mother, father, and adolescents):

*Adapted Healthy Eating Index (AHEI)*: An adaptation of the U.S. Healthy Eating Index (HEI) was used, developed by Kennedy et al. [39] and translated by Norte and Ortiz [40] for the Spanish-speaking population. This version was previously used by the Chilean Ministry of Health to measure the overall quality of food in the Chilean population [41]. The AHEI is comprised of nine food groups and diet variety. The first four variables correspond to foods that should be consumed on a daily basis, five and six correspond to foods that should be consumed weekly, while seven, eight and nine are foods that should be consumed occasionally, and 10 refers to diet variety, a fundamental goal in healthy eating [40]. For the first nine variables, respondents indicated their consumption frequency of the target food. Each variable received a score, ranging from 0 to 10, according to the degree of compliance with dietary recommendations (see the criteria in 46). The last variable, relating to diet variety, is constructed using the consumption frequency of the nine target foods: 2 points were received if the respondent complied with each of the daily recommendations and 1 point was received if he/she complied with each of the weekly recommendations. The AHEI score was calculated by adding the score obtained in each of the variables, allowing for a maximum of 100 points. According to this index, scores above 80 are indicative of a “healthy” diet; scores between 51 and 80 correspond to a diet that “requires changes”; scores below 50 correspond to “unhealthy” diets [39].

*Satisfaction with Life Scale (SWLS)* [42]: This scale consists of five items, grouped into a single dimension, which evaluate overall cognitive judgments about a person’s own life (e.g., “*In most ways my life is close to my ideal*”). The Spanish-language version of the SWLS was used, which has shown good internal consistency in previous studies with adults in Chile [32,33,34]. In the present study, the SWLS showed a good level of internal consistency for all family members (Cronbach’s α mothers = 0.903, fathers = 0.895, children = 0.913). The SWLS scores were calculated as the sum of the five scale items (higher scores indicate higher satisfaction with life).

*Satisfaction with Food-related Life (SWFoL)* [30]: This scale consists of five items, grouped in a single dimension, that evaluate a person’s overall assessment regarding their food and eating habits (e.g., “*Food and meals are positive elements*”). The Spanish-language version of the SWFL was used in the present study, which has shown good internal consistency in previous cross-sectional and longitudinal studies with adults in Chile [32,33,34,43]. The SWFoL showed a good internal consistency for all family members (Cronbach’s α mothers = 0.857, fathers = 0.766, children = 0.910). The SWFoL score was calculated as the sum of the five scale items (higher scores indicate higher satisfaction with food-related life).

*Satisfaction with Family Life (SWFaL) scale*. This scale, proposed by Zabriskie and McCormick [44], is an adaptation of the SWLS [42], in which the words “family life” replaced the word “life” in each of the five original items of the SWLS. Family satisfaction can be defined as a conscious cognitive judgment of one’s family life based on the subjective criteria of each individual [45]. The SWFaL has shown good internal consistency in previous studies in the U.S., Canada, UK, Australia, and New Zealand family samples, with five items grouped in a single dimension [45]. This study used the Spanish-language version of the SWFaL, which showed good internal consistency in a previous study with undergraduate students in Chile [46]. The SWFaL showed a good level of internal consistency (Cronbach’s α mothers = 0.928, fathers = 0.917, children = 0.925). The SWFaL score was calculated as the sum of the five scale items (higher scores indicate higher satisfaction with food-related life).

In addition, both parents and their children were asked to rate the importance of food for their well-being. Parental nutrition knowledge on dietary recommendations was measured using two dimensions, based on the proposal by Grunert et al. [47]. The first dimension consisted of 12 items measuring perceptions on whether health experts recommend that one should eat more, about the same, less or try to avoid a series of nutrients. The second dimension consisted of seven items measuring perceptions on whether health experts recommend that one should eat a lot, some, a little, or try to avoid different food groups. In both dimensions, responses were coded as correct or incorrect. When participants selected the option “try to avoid”, responses were coded as correct even when the correct answer would have been “eat less”. Nutrition knowledge for each dimension was estimated as the number of correct responses.

Mothers were asked about the number of family members, number of children, number of days per week that all family members eat together during the week, and the number of days per week that they eat homemade food, buy ready-to-eat food, order food at home, or eat at restaurants or fast-food outlets. In addition, families were asked to indicate their monthly expenditure on food as well as to identify the person responsible for deciding what is eaten and what food is purchased for the household. All family members were asked to indicate their ethnic origin and their own approximate weight and height, in order to determine body mass index (BMI, kg/m^2^). Education level and occupation of the head of household were used to determine socioeconomic status (SES). These variables are conceptually related to income and cultural level, allowing a simple proxy of the socioeconomic status of Chilean households [48]. Mothers, fathers, and children were asked about the places they eat when they do not eat at home with their family. For more detailed information on the instruments and questions, see the Appendix.

A pilot test of the questionnaires was conducted with 10 families with the same characteristics as the sample, following the same recruitment method. As the pilot test of the instrument was satisfactory, no changes were required for the questionnaires or the interview procedure.

### 2.3. Data Analysis

#### 2.3.1. Descriptive Statistics

Descriptive statistics were used for each of the variables. Frequency distributions were obtained, and the mean and standard deviation were calculated for continuous variables. The correlation between responses of the three family members for AHEI was calculated.

#### 2.3.2. Reliability and Validity of the Satisfaction with Life, Satisfaction with Food-Related Life and Satisfaction with Family Life Scales

Given that there are currently no studies in the literature that simultaneously combine the SWLS, SWFoL, and SWFaL in adults and adolescents, the psychometric properties of these scales and the relationship between life satisfaction, satisfaction with family life, and satisfaction with food-related life were assessed using confirmatory factor analysis (CFA) with correlated latent constructs for each family member. In addition, a CFA with correlated latent constructs was carried out to assess the relationships between the SWLS, SWFoL, and SWFaL among the three family members. Analysis was implemented using LISREL 8.8 (Scientific Software International, Inc. Chicago, IL, USA, 2007). Parameters were estimated by robust maximum likelihood [49]. In terms of construct validity, convergent validity was assessed by inspecting the standardized factor loadings of each scale (ideally > 0.5) as well as their significance, composite reliability (values > 0.7) and average variance extracted (AVE, values > 0.5) [49]. Discriminant validity was obtained by comparing the AVE for each construct with the square of the correlation between the scales [50]. Various indicators were used to evaluate the goodness of fit of the models: the comparative fit index (CFI), goodness-of-fit index (GFI), adjusted goodness-of-fit index (AGFI) and the root mean square error of approximation (RMSEA). A model fits reasonably well if CFI, GFI, and AGFI are greater than 0.90 and if the RMSEA is below 0.08 [51].

#### 2.3.3. Identification of Families with Different Diet Quality

In order to identify families with different profiles in terms of family member diet quality, a cluster analysis was used on the AHEI scores of each family member, considering squared Euclidean distances and Ward’s criteria [49]. The number of clusters was determined considering the percentage change in the recomposed conglomeration coefficients. To define segments, Pearson’s Chi^2^ test was employed to the discrete variables and an analysis of variance for the continuous variables. Given that the Levene’s statistic indicated non-homogeneous variances in each continuous variable observed, the variables for the analysis of variance that resulted in significant differences were subjected to Dunnett's T3 multiple comparisons test. Data were analyzed using SPSS for Windows v. 23.0（IBM Corp. IBM SPSS Statistics for Windows, Armonk, NY, USA.

## 3. Results

### 3.1. Study Participants

A total of 300 two-parent families participated in the study. Only one adolescent per family was interviewed, resulting in a total of 300 responses for mothers, fathers, and adolescents. Table 1 shows the demographic characteristics of the sample, the three subsample compositions according to body mass indexes following the criteria of the World Health Organization [1] for mothers and fathers and the criteria of the World Health Organization [52] and the Technical Norm of Nutritional Evaluation of children from five to 19 years old of the Ministry of Health of Chile [53]. Table 1 also includes family eating habits, monthly expenditure on food, and the person responsible for deciding what to eat and what food to purchase for the household.

### 3.2. Diet Quality, Eating Habits, and Nutritional Knowledge

The average AHEI score for the family members was similar, and denoted diets that required changes: 64.5 (SD = 14.3) for mothers, 58.9 (SD = 14.6) for fathers, and 62.0 (SD = 15.0) for adolescents. The AHEI scores of the family members were significantly correlated, but the highest correlation coefficient was found for the AHEI scores of mothers and adolescents. The Pearson correlation coefficients corresponded to 0.447 (*p* < 0.01) for mothers and adolescents, 0.308 (*p* < 0.01) for fathers and mothers, and 0.215 (*p* < 0.01) for fathers and adolescents.

According to the cut-off proposed by Kennedy et al. [39], the minority of family members had a healthy diet that did not require changes. In the case of mothers, 14.7% had a healthy diet, 69.7% had diets that required changes, and 15.7% had an unhealthy diet. Regarding fathers, only 5.3% had a healthy diet, 69.7% required changes, and 25.0% had an unhealthy diet. Similarly, 11.3% of the children had a healthy diet, 67.3% required changes, and 21.3% had an unhealthy diet. The proportion of participants who had a healthy diet was significantly higher in the subsample of mothers than in the subsamples of fathers and adolescents (*p* ≤ 0.001). In addition, the proportion of participants who had an unhealthy diet was significantly higher in the subsample of fathers than in the mother and children subsamples (*p* ≤ 0.001).

Table 2 shows the consumption frequency of the food categories included in the AHEI for mothers, fathers and adolescents. As can be seen, mothers consumed fruit and vegetables significantly more often than fathers and adolescents. Fathers showed the highest consumption frequency of sausages and cold meats, whereas adolescents showed the highest consumption frequency of cereals and derivatives, milk and dairy products, and sweets. Fathers and children showed a higher consumption frequency of soda than mothers. Family members did not significantly differ in their consumption frequency of meat and legumes (*p* > 0.1).

Average nutrition knowledge on dietary recommendations related to nutrient intake was 69.1% for mothers and 66.0% for fathers. Regarding average nutrition knowledge on dietary recommendations related to food intake, it was 65.3% for mothers and 64.9% for fathers. In addition, 33.7% of the mothers considered food to be considerably important for their well-being and 31.3% considered it to be very important. For fathers, these percentages were 35.0% and 31.3%, respectively. In the case of adolescents, 35.0% considered food to be very important for their well-being and 34.7% considered it to be considerably important.

### 3.3. Reliability and Validity of the Satisfaction Scales

Reliability and validity of the satisfaction scales were evaluated using CFA. Results indicated that the SWLS, SWFoL and SWFaL scales satisfied the composite reliability test (above 0.7) for the subsamples of mothers, fathers, and children. The scales also satisfied the AVE values (higher than 0.5) in the three subsamples (mothers, fathers, and adolescents) (Table 3). The value of the squared correlation between the SWLS and SWFoL was lower than the AVE of the scales, which verified the discriminant validity between the constructs in the three subsamples. The discriminant validity between SWLS and SWFaL and between SWFoL and SWFaL was also verified in the three subsamples [50].

All the factor loadings from the SWFoL, SWFaL, and SWLS were over 0.58 and statistically significant in the three subsamples. Therefore convergent validity was shown for the three scales and the internal validity of the measurement models was adequate in the three subsamples.

Regarding the CFA with correlated latent constructs for each family member, the models had a good fit of the data in the subsample of mothers (RMSEA = 0.057, CFI = 0.99, GFI = 0.94, AGFI = 0.92), fathers (RMSEA = 0.059, CFI = 0.99, GFI = 0.93, AGFI = 0.90), and children (RMSEA = 0.052, CFI = 0.99 GFI = 0.95, AGFI = 0.93). The correlation values between SWLS, SWFaL, and SWFoL were positive and significant in the three subsamples (Table 3).

Regarding CFA with correlated latent constructs between the SWLS, SWFoL, and SWFaL among the three family members, the CFA model showed a good fit of the data for the SWFoL (RMSEA = 0.055, CFI = 0.98, GFI = 0.93, AGFI = 0.91). Correlation values between the SWFoL for mothers, fathers and adolescents were positive and significant (Table 4). The CFA model also showed a good fit of the data for the SWFaL (RMSEA = 0.056, CFI = 0.98, GFI = 0.94, AGFI = 0.92). The correlation values between the SWFaL of mothers, fathers, and adolescents were positive, but only significant between mothers and fathers and between mothers and adolescents. In the same way, the CFA model also showed a good fit of the data for the SWL scale (RMSEA = 0.055, CFI = 0.98, GFI = 0.93, AGFI = 0.91). Correlation values between mothers, fathers, and adolescents were positive, but again only significant between mothers and fathers and between mothers and adolescents. Therefore, the results indicate that adolescents’ life satisfaction, satisfaction with food-related life and satisfaction with family life were more strongly correlated with those of their mothers than with those of their fathers. In parallel, overall life satisfaction and satisfaction in the family and food domains were positively correlated for both parents.

Table 5 shows the mean SWLS, SWFoL, and SWFaL scores for mothers, fathers, and children. The AHEI was significantly and positively correlated with SWLS, SWFoL, and SWFaL for mothers and fathers, whereas in the case of adolescents, AHEI scores were only significantly correlated with SWLS and SWFoL. Correlation coefficients between AHEI and satisfaction scores were higher for mothers than for fathers and children. In particular, the highest coefficients were found for the correlation between AHEI and SWFoL for mothers.

### 3.4. Profiles of Families with Different Diet Quality

Cluster analysis identified three groups of families with different diet qualities. Families in Group 1 (39.3% of families) were characterized by the lowest AHEI scores for all family members, indicating the lowest diet quality (Table 6). The average AHEI scores corresponded to unhealthy diets for adolescents and diets that required changes for both parents. Families in Group 2 showed the highest AHEI average scores, which corresponded to healthy diets for mothers and adolescents and to diets that required changes for fathers. Finally, families in Group 3 were characterized by intermediate average AHEI scores that corresponded to diets that required changes for all family members. Within Group 1, the average AHEI score for mothers (*p* ≤ 0.001) and fathers (*p* ≤ 0.05) were significantly higher than the average AHEI score for children. Within Groups 2 and 3, the average AHEI score for mothers and children (*p* ≤ 0.001) were significantly higher than the average AHEI score for fathers.

Differences in diet quality between the three groups were explained by differences in the consumption frequency of most of the food groups, except for legumes in the three family members, and for meat in the subsamples of fathers and children (see Supplementary Tables S1–S3). All family members in Group 1 had a lower consumption frequency of fruit, vegetables, cereals and derivatives, and milk and dairy products than those in Groups 2 and 3, and a higher consumption frequency of soft drinks with sugar, sausages, and cold meats. On the contrary, family members in Group 2 had the highest consumption frequency of healthy foods (fruit, vegetables, cereals and derivatives, milk and dairy products) and the lowest consumption frequency of unhealthy foods (soft drinks with sugar, sausages, and cold meats). In addition, family members in Group 2 had the highest average diet variety scores, followed by Group 3 and finally Group 1 (*p* ≤ 0.001) (Table 6).

Regarding eating habits, as shown in Table 6, family members in Group 2 had breakfast together significantly more often than family members from Groups 1 and 3 (*p* ≤ 0.001). They also had lunch together more often than family members from Group 1 (*p* ≤ 0.05). Moreover, families in Group 2 ate homemade foods significantly more often per week than families in Group 1 and 3 (*p* ≤ 0.05). On the contrary, families in Group 1 bought ready-to-eat foods and ate at fast food outlets (*p* ≤ 0.05) significantly more often than families in Groups 2 and 3. The average monthly expenditure on food of families from Group 2 was significantly higher than that from Groups 1 and 3 (*p* ≤ 0.05).

As shown in Table 6, the average SWLS, SWFoL and SWFaL scores of mothers from Group 2 were significantly higher than in Groups 1 and 3 (*p* ≤ 0.05). In addition, the average SWLS and SWFaL scores of fathers from Group 2 were significantly higher than in Groups 1 and 3 (*p* ≤ 0.05).

Group 2 was composed of a greater proportion of families in which the mother decided what food to purchase for the household (*p* ≤ 0.05), whereas Group 3 had a greater proportion of families in which the purchasing decisions were made by both parents (Table 7). Group 1 had a higher proportion of obese mothers (*p* ≤ 0.05). Additionally, Group 1 had greater proportions of mothers and children who consider food to be slightly important for their well-being (*p* ≤ 0.05) and Group 2 had greater proportions of mothers and children who consider food to be completely important for their well-being.

Finally, no significant differences between the groups were found for the importance that fathers attached to food for their well-being, parental nutrition knowledge on dietary recommendations, the number of family members, number of children, number of siblings, sex of the interviewed adolescent, ethnic origin, the number of days a week they ate dinner together, number of days they ordered takeout food, the person responsible for purchasing foods, fathers and adolescents’ BMI, socioeconomic status, the place family members ate when they did not eat at home with their family, fathers’ scores on the SWFoL scale, and children’s scores on the SWLS, SWFoL, and SWFaL scales (*p* > 0.1).

## 4. Discussion

This study assessed diet quality, satisfaction with life, family life, and food-related life in mother–father–adolescent triads and identified family groups according to family member diet quality. Findings derived from this study contribute to the development of strategies for mitigating unhealthful eating habits and their negative consequences for family members’ physical and psychological well-being.

### 4.1. Diet Quality of Mothers–Fathers–Adolescents

Parents can influence their children’s eating behavior by providing (un)healthful foods at home [9,12,13,14] or modeling (un)healthful foods choices. Average AHEI scores evidenced the need to make changes in the diets of the majority of the families, which is in agreement with the results reported by the Chilean Ministry of Health in 2014 [41]. There was a relatively low compliance with recommended dietary guidelines. Consumption frequency of fruit, vegetables, cereals and derivatives, and milk and dairy products was below the recommendations, whereas consumption of ultra-processed foods, such as sausages and cold meats, sweets, and sweetened soft drinks was above the recommendations.

The proportion of participants with healthy diets was higher in the subsamples of mothers and adolescents in comparison with the subsample of fathers, whereas the opposite trend was found for the proportion of participants with unhealthy diets. This finding is in line with studies carried out in Australia [17,18] and in European countries [13], which suggest that mothers exert a positive influence on their children’s food consumption because they are more likely than fathers to adhere to dietary guidelines. In this regard, although AHEI scores were significantly and positively correlated for all family members, the highest correlation coefficients were found for mothers and adolescents. These results confirm the positive influence that mothers have on their children’s HEI score and eating behavior [18,19] as well as on their husbands’ food consumption [18].

It has been suggested that parental influences on children’s food habits may be related to specific food groups [13,17]. Studies conducted in Australia and the USA have found that mothers mainly influence their children’s overall diet quality score and their consumption of healthy foods, such as vegetables, fruits, and dairy products [13,17,18]. On the contrary, the influence of fathers on their children’s intake is mainly related to foods that are high in sugar, fat and saturated fat, like sweets, and unhealthy beverages [13,17]. In the present work, the fruit consumption frequency for adolescents was more similar to the consumption frequency of mothers than that of fathers, whereas the opposite trend was found for vegetables and sugar-sweetened soft drinks. In addition, the consumption frequency of milk and dairy products and sweets was higher for adolescents than for parents. This result may be related to the consumption of these products outside of the home or even at home.

### 4.2. Profiles of Families with Different Diet Quality

Three family profiles were identified: Group 1 corresponded to “families with an unhealthy diet” (39.3%), Group 2 “mothers and adolescents with healthy diets, whereas fathers’ diet required changes” (14.3%), and Group 3 “families that required changes in their diet” (46.4%). The percentage of families with unhealthy diets was higher than that reported in the survey conducted by the Chilean Ministry of Health in 2014, which reported that 5% of the Chilean population had a healthy diet, 87% required changes and 8% had an unhealthy diet [41].

The AHEI scores from family members of the three groups confirm the similarities between the dietary patterns of parents and their children, which is in agreement with results reported by Hebestreit et al. [13]. However, it is interesting to highlight that fathers obtained lower AHEI scores than mothers in the three groups. This difference was particularly relevant in the group with the healthiest dietary pattern (Group 2), where the AHEI scores of the fathers indicated that their diet required changes, while the diet of mothers and adolescents could be regarded as healthy. One possible explanation for this result is that males tend to be less motivated with regards to their eating behavior in comparison to females [54], even if their spouses promote healthy eating habits at home [18]. In this sense, previous studies have shown that males have poorer diet quality than females [55,56,57].

Differences in the AHEI mean scores of family members from Groups 1 and 2 were related to differences in their eating habits. Families in Group 2 (who exhibited the highest AHEI scores) reported that they had breakfast and lunch together more often per week than Group 1 (who showed the lowest AHEI scores). These results are consistent with previous studies that reported a positive association between frequent family meals and a healthier diet in adolescents [9,13,22,23,24]. Families from Groups 1 and 3 did not differ in the average number of days in which family members have breakfast and lunch together; however, the AHEI scores of Group 3 family members were higher than for Group 1. Therefore, it seems that frequent family meals are not enough to improve family member diet quality; it is also necessary for parents to provide healthful foods at home [9,12,14], to model healthy eating practices [8,9] and to encourage healthful food choices [9]. Healthier parenting practices may explain adolescents’ higher consumption of fruit and vegetables in Group 3 in comparison to Group 1, which is in agreement with the results reported by Watts et al. [9].

Differences in AHEI scores between the groups were also related to differences in the source of family meals, which has also been reported to be important for maintaining a healthy diet and weight [58]. Families in Group 2, with the highest AHEI scores, reported that they ate homemade foods a higher number of days than the other two groups. On the contrary, families in Group 1 reported buying ready-to-eat food and eating at fast food outlets more frequently than the other two groups. Previous studies have reported that diet quality is positively associated with a greater frequency of cooking and eating at home [25] and a lower frequency of eating out, particularly at fast food restaurants [11,25,58,59,60,61].

Current literature suggests that one individual normally makes most of the decisions about food purchases and preparation for the entire family [18], impacting the eating habits of each family member [62]. The present work showed similar results as mothers were the main person responsible for making dietary choices within families. Therefore, the higher AHEI scores from the three family members from Group 2 may be related to the healthy food choices of the mothers in this group. Similar results were reported by Rhodes et al. (2016) in Australia. These authors found that mothers influenced fruit and vegetable intake by controlling purchasing decisions (e.g., by shopping for food or editing family grocery shopping lists). On the contrary, mothers in Group 1 did not seem to positively influence the consumption of healthy food as they provided an unhealthy food environment for their family [23,63,64] and introduced negative parental modeling to their adolescent children [13,65].

The difference in food decisions between mothers from Groups 1 and 2 was not related to their nutrition knowledge. These results are congruent with a study conducted in the USA in which no association was observed between adolescents’ and mothers’ nutrition knowledge and diet quality [66]. One possible explanation is that possessing nutrition knowledge does not necessarily translate into healthy eating behaviors [11,19,67,68]. In fact, differences in the food choices of mothers in Groups 1 and 2 may be related to the level of importance that they attach to foods for their well-being. In fact, positive parental modeling and a healthy food environment have been associated with a greater interest in food [69]. The latter is consistent with the higher proportion of mothers from Group 2 who considered food to be completely important to their well-being when compared to mothers in Group 1. It is important to highlight that mothers also seem to model the importance they assign to food as related to well-being for their children, since most children from Group 2 considered food to be completely important to their well-being, with the opposite behavior in the children from Group 1. Our findings are consistent with those reported by Schnettler, Denegri et al. [32], and Schnettler, Miranda et al. [33] in Chilean university students. These authors found that students who assign more importance to food for their well-being have healthier eating habits. In this regard, Thornton et al. [70] suggested that healthy behaviors are influenced by an individual’s ability to make healthy behavior choices and his/her motivation to do so.

Contrary to what was expected, family profiles did not differ according to their SES, as previously reported by several authors, e.g., [25,71,72,73,74]. Nevertheless, findings from the present study are in line with research that shows that higher HEI scores are associated with higher food expenditure [56,75], which is associated with the higher relative cost of healthy foods [14,75,76,77]. One possible explanation for the lack of statistical differences in the family SES profiles in this study may be related to the variables that were considered related to family SES, namely education and occupation of the head of the household [48], without considering household income. Nevertheless, the average monthly expenditure on foods of Group 2 was close to the average monthly expenditure on foods of the higher income quintile in Chile. Meanwhile, monthly food expenditure of Groups 1 and 3 were close to the second higher income quintile in Chile according to the results of the VII Chilean Survey of Family Budgets [78]. Therefore, it is possible to suggest that families from Group 2 spend more per month on food, particularly on healthy and expensive foods (vegetables, fruits and milk and dairy products) than Groups 1 and 3 due to their higher income. Another possible explanation may be related to whether families have enough economic resources, and whether parents had sufficient time [14,54], motivation, and skilled labor to improve their eating behaviors [54].

### 4.3. Relationship between Diet Quality and Satisfaction with Life, Satisfaction with Family Life, and Satisfaction with Food-Related Life

It is noteworthy that findings from the present work expand upon existing research regarding family influence on adolescents’ eating behaviors beyond developed countries. This study is also the first to relate diet quality to overall life satisfaction, satisfaction with food-related life, and satisfaction with family life in mother–father–adolescent children triads.

Correlation values over 0.5 indicated a high level of relationship [79] between the SWFoL and SWLS in all three family members, confirming the positive relationship between satisfaction with food-related life and life satisfaction reported with adults [31,32,33,34,35]. It is also noteworthy to find this positive and high correlation in adolescents as well. The higher correlation values between SWFaL and SWLS in the three family members also indicate a strong relationship between these constructs [79]. This finding is in line with studies that indicate that good family relationships positively influence a person’s life satisfaction in general [80,81].

The correlation between SWFoL and SWLS was lower than the correlation between SWFaL and SWLS between the three family members. Pavot and Diener [82] argued that, although there may be some general agreement on the components of high levels of well-being, individuals are likely to assign different weights to each component [45]. Therefore, results indicate that the family domain would be more important than the food domain for overall life satisfaction for both parents and adolescents [11,64].

The significantly higher SWLS and SWFoL scores of the mothers from Group 2 families (highest AHEI scores) compared to mothers from Groups 1 and 3 confirm the relationship between greater levels of life satisfaction, satisfaction with food-related life and better eating habits [32,33]. Conversely, the higher AHEI and SWLS scores of fathers from Group 2 and the lower scores of fathers from Groups 1 and 3 confirm the relationship between greater levels of life satisfaction and better eating habits. Nevertheless, the relationship between higher levels of satisfaction with food-related life and better eating habits was not confirmed in the subsample of fathers and adolescents. Further research is required to understand the relationship between eating patterns and satisfaction with food-related life, particularly in adolescents.

One remarkable finding is that mothers and fathers from Group 2 had significantly higher levels of life satisfaction and satisfaction with family life than those from Groups 1 and 3, which may be associated with the differences in the frequency of family meals between groups. There is evidence that family meals may lead to more connected and communicative families, which in turn may lead to more positive experiences [9], increasing the well-being of family members given the positive association with social support [27,32,35].

### 4.4. Limitations of the Study

One of the main limitations of this study is its cross-sectional design. Another limitation is related to the non-probabilistic nature of the sample and its relatively small size, as well as having been conducted with families from only one city in one country, which does not permit generalization of the results. Responses may be affected by social desirability as data were self-reported. Indeed, self-reported eating habits may be affected by an overestimation of the frequency of healthy food consumption and an underestimation of the frequency of unhealthy food consumption, as reported in previous studies [73]. In addition, although AHEI may be a useful tool to measure diet quality, it does not include all possible food groups and the quantity of food consumed is not assessed.

## 5. Conclusions

Satisfaction with food-related life and satisfaction with family life have positive relationships with life satisfaction of different family members, and both life domains are positively related. Since the findings of this study show that better diet quality and more frequent family meals seem to be related to higher levels of satisfaction with life and family life in parents, studies on the relationship between food and family should not only consider diet-related variables, but also psychological variables. Similar research should be conducted on single-parent households with adolescent children. A special emphasis should be placed upon the detection of variables that lead some mothers to have unhealthy diets, provide an unhealthy food environment for their family, and introduce negative parental modeling to their adolescent children. In addition, future research should identify variables related to satisfaction with food-related life in fathers and adolescents.

The findings of the present study allow us to make suggestions about intervention methods to improve overall family diet quality according to each profile’s characteristics, as well as to enhance family member diet quality. In addition, the findings of this study suggest that interventions that aim to improve levels of satisfaction with food-related life and satisfaction with family life may also boost life satisfaction for all family members.

## Figures and Tables

**Table 1:** Sample characteristics.

Characteristic	Total Sample (*n* = 300)
Mother age [Mean (SD)]	41.6 (6.8)
Father age [Mean (SD)]	44.1 (7.2)
Children age [Mean (SD)]	13.2 (2.3)
Number of family members [Mean (SD)]	4.4 (1.0)
Number of children [Mean (SD)]	2.4 (1.0)
Number of siblings [Mean (SD)]	1.4 (1.0)
Children sex (%)	
Female	48.7
Male	51.3
Socioeconomic status (%)	
High and upper-middle	17.0
Middle	18.7
Lower-middle	35.0
Low	24.0
Very low	5.3
Father’s ethnic origin (%)	
Mapuche *	14.9
Non-Mapuche	85.1
Mother’s ethnic origin (%)	
Mapuche	19.4
Non-Mapuche	80.6
Child’s ethnic origin (%)	
Mapuche	28.9
Non-Mapuche	71.1
BMI of fathers [Mean (SD)]	28.3 (4.0)
BMI of mothers (%)	
Normal range (18.5–24.9)	24.0
Overweight (25.0–29.9)	42.7
Obesity (≥30)	33.3
BMI of fathers (%)	
Normal range (18.5–24.9)	17.7
Overweight (25.0–29.9)	55.7
Obesity (≥30)	26.7
BMI of children (%)	
Undernourished (≤−2 SD)	6.3
Underweight (≤−1 to −1.9 SD)	13.7
Normal range (+0.9 to −0.9 SD)	53.7
Overweight (≥+1 to +1.9 SD)	19.7
Obese (≥+2 SD)	6.7
Number of days a week families eat together [Mean (SD)]	
Breakfast	4.1 (3.2)
Lunch	4.4 (2.5)
Dinner	3.8 (3.2)
Number of days families eat different types of foods [Mean (SD)]	
Homemade foods	6.4 (1.4)
Buy ready-to eat food	0.4 (1.0)
Order food at home	0.3 (0.6)
Eat at restaurants	0.4 (0.7)
Eat at fast-food outlets	0.3 (0.6)
Person who decides the purchasing of food (%)	
Mother	67.9
Both parents	25.3
Father	4.8
All (mother, father, and children)	1.4
Grandmother	0.7
Person who purchases food (%)	
Mother	54.1
Both parents	35.7
Father	7.5
Mother with a child	1.7
All (mother, father, and children)	0.7
Grandmother	0.3
Monthly food expenditure [Mean (SD)]	274.2 (129.4)

* Mapuche is the main indigenous group in Chile.

**Table 2:** Consumption frequency of the foods included in the Adapted Healthy Eating Index (AHEI) for mothers, fathers, and adolescents.

Food	Mothers	Fathers	Children
Cereals and derivatives		*p* = 0.000	
Daily consumption	20.3	16.0	29.3
Three or more times a week, but not daily	21.0	20.3	26.7
Once or twice a week	30.7	22.0	25.0
Less than once a week	10.7	13.7	6.7
Never or almost never	17.3	28.0	12.3
Vegetables		*p* = 0.000	
Daily consumption	58.7	49.7	43.0
Three or more times a week, but not daily	29.0	31.3	33.3
Once or twice a week	10.7	15.7	17.3
Less than once a week	1.0	3.0	2.7
Never or almost never	0.7	0.3	3.7
Fruit		*p* = 0.001	
Daily consumption	46.0	27.0	37.0
Three or more times a week, but not daily	30.3	38.0	29.3
Once or twice a week	16.7	22.0	24.0
Less than once a week	6.0	9.7	7.7
Never or almost never	1.7	3.3	2.0
Milk and dairy products		*p* = 0.000	
Daily consumption	35.0	28.0	58.3
Three or more times a week, but not daily	28.0	22.7	18.0
Once or twice a week	22.0	25.3	13.3
Less than once a week	8.7	14.3	6.3
Never or almost never	6.3	9.7	4.0
Meat		*p* = 0.264	
Daily consumption	14.3	18.7	17.3
Three or more times a week, but not daily	43.3	41.0	37.3
Once or twice a week	31.7	33.0	34.3
Less than once a week	9.0	5.3	7.0
Never or almost never	1.7	2.0	4.0
Legumes		*p* = 0.621	
Daily consumption	1.7	2.3	3.0
Three or more times a week, but not daily	13.0	9.7	15.0
Once or twice a week	57.0	59.7	55.7
Less than once a week	20.0	18.3	17.0
Never or almost never	8.3	10.0	9.3
Sausages and cold meats		*p* = 0.006	
Daily consumption	12.7	19.0	10.0
Three or more times a week, but not daily	21.7	23.7	27.7
Once or twice a week	23.0	24.3	27.3
Less than once a week	22.7	22.0	19.0
Never or almost never	20.0	11.7	16.0
Sweets		*p* = 0.000	
Daily consumption	7.3	9.3	18.3
Three or more times a week, but not daily	19.0	17.0	25.7
Once or twice a week	25.3	18.7	24.7
Less than once a week	22.3	28.0	18.0
Never or almost never	26.0	27.0	13.3
Soft drinks with sugar		*p* = 0.001	
Daily consumption	22.3	29.0	30.3
Three or more times a week, but not daily	14.0	20.7	20.7
Once or twice a week	18.7	15.3	19.7
Less than once a week	16.7	16.0	14.7
Never or almost never	28.3	19.0	14.7

*p* value corresponds to the (bilateral) asymptotic significance obtained in Pearson’s chi-squared test.

**Table 3:** Composite reliabilities, average variance extracted (AVE), correlations, and squared correlations between the Satisfaction with Food-related Life scale (SWFoL), Satisfaction with Family Life (SWFaL) scale, and Satisfaction with Life Scale (SWLS) in mothers, fathers, and children.

Family Member/Scale	Composite Reliability	AVE	SWFoL	SWFaL	SWLS
Mothers					
SWFoL	0.863	0.557	-	0.303	0.270
SWFaL	0.930	0.727	0.55	-	0.547
SWLS	0.903	0.653	0.52	0.74	-
Fathers					
SWFoL	0.833	0.501	-	0.360	0.292
SWFaL	0.921	0.699	0.60	-	0.578
SWLS	0.902	0.651	0.54	0.76	-
Children					
SWFoL	0.902	0.647	-	0.348	0.336
SWFaL	0.924	0.710	0.59	-	0.620
SWLS	0.918	0.692	0.58	0.79	-

Values over diagonal in mothers, fathers and children subsamples indicate squared correlations between constructs. Values under diagonal in mothers, fathers and children subsamples indicate correlations between constructs.

**Table 4:** Correlations between the Satisfaction with Food-related Life scale (SWFoL), Satisfaction with Family Life (SWFaL) scale, and Satisfaction with Life Scale (SWLS) between the three family members.

Scale/Family Member	Mother	Father
SWFoL		
Mother	-	
Father	0.58	-
Children	0.22	0.14
SWFaL		
Mother	-	
Father	0.44	-
Children	0.25	0.11
SWLS		
Mother	-	
Father	0.39	-
Children	0.18	0.11

**Table 5:** Average scores of the Satisfaction with Food-related Life scale (SWFoL), Satisfaction with Family Life (SWFaL) scale, and Satisfaction with Life Scale (SWLS) of each family member, and correlation with the Adapted Healthy Eating Index (AHEI).

	Mothers	Fathers	Children
Scale			
SWLS	23.8 (SD = 4.8)	24.2 (SD = 4.5)	24.3 (SD = 5.5)
SWFoL	22.8 (SD = 4.7)	22.9 (SD = 4.5)	22.8 (SD = 6.2)
SWFaL	24.4 (SD = 4.9)	24.7 (SD = 4.8)	24.5 (SD = 5.6)
Correlation coefficients AHEI-SWLS AHEI-SWFoL AHEI-SWFaL	0.261 ** 0.293 ** 0.232 **	0.220 ** 0.176 ** 0.209 **	0.132 * 0.148 * 0.088 ns

** *p* < 0.01. * *p* < 0.05. ns: non-significant.

**Table 6:** Characterization of the three groups of families with different diet quality, identified using hierarchical cluster analysis.

Component	Group 1 (*n* = 118)	Group 2 (*n* = 43)	Group 3 (*n* = 139)	F	*p*-Value
*AHEI*					
Mother	54.9 c	82.8 ^a^	67.1 ^b^	111.204	0.000 **
Father	52.0 c	69.4 ^a^	60.8 ^b^	32.235	0.000 **
Adolescents	48.5 c	82.5 ^a^	67.1 ^b^	264.850	0.001 **
*AHEI variety score*					
Mother	2.1 c	7.1 ^a^	4.3 ^b^	57.327	0.000 *
Father	2.6 c	4.7 ^a^	3.8 ^b^	17.465	0.000 *
Children	2.2 c	7.3 a	5.0 ^b^	131.152	0.000 *
*Number of days a week families eat together*					
Breakfast	3.49 b	5.21 ^a^	4.41 ^b^	6.985	0.001 **
Lunch	3.93 b	5.30 ^a^	4.55 ^a,b^	4.993	0.007 **
*Number of days families eat different types of foods*					
Homemade foods	6.23 b	6.86 ^a^	6.43 ^b^	3.123	0.045 *
Buy ready-to eat food	0.71 a	0.21 ^b^	0.40 ^b^	5.330	0.005 *
Eats at fast food outlets	0.42 a	0.14 ^b^	0.21 ^b^	5.648	0.004 *
*Monthly expenditure on food ($US)*	264.18 b	329.75 ^a^	273.98 ^b^	4.803	0.009 *
*SWLS*					
Mother	23.9 b	25.7 ^a^	23.4 ^b^	3.959	0.040 *
Father	23.7 b	25.7 ^a^	23.9 ^b^	3.193	0.042 *
*SWFoL*					
Mother	22.4 b	25.1 ^a^	22.6 ^b^	4.122	0.027 *
*SWFaL*					
Mother	23.5 b	25.9 ^a^	23.2 ^b^	3.589	0.032 *
Father	24.3 b	26.6 ^a^	24.4 ^b^	3.950	0.020 *

* Significant at 5%. ** Significant at 1%. Different letters in the line indicate significant differences according to Dunnett’s T3 multiple comparisons test (*p* ≤ 0.05).

**Table 7:** Differences between the three groups of families with different diet quality in terms of the person who decided to buy food, body mass index (BMI) of the mother, and the importance that the mother and adolescents placed on food for their well-being.

	Group 1 (*n* = 118)	Group 2 (*n* = 43)	Group 3 (*n* = 139)
*Person who decides to buy food*		*p* = 0.040	
Both parents	22.0	14.0	30.9
Mother	70.7	82.9	61.0
Father	6.0	0.2	5.0
All (mother, father, and children)	0.4	0.4	2.9
Grandmother	0.9	2.4	0.1
*BMI of mother*		*p* = 0.004	
Normal range	16.9	34.9	26.6
Overweight	37.3	51.2	44.6
Obesity	45.8	14.0	28.8
*Mother: importance of food for well-being*		*p* = 0.017	
Hardly important	2.5	0.1	2.9
Slightly important	39.8	0.1	2.9
Very important	4.2	18.4	28.1
Considerably important	28.8	30.2	38.8
Completely important	24.6	51.2	27.3
*Children: importance of food for well-being*		*p* = 0.043	
Not at all important	0.1	0.1	0.7
Hardly important	0.1	0.1	1.4
Slightly important	28.8	0.1	7.9
Very important	10.0	9.3	19.4
Considerably important	33.1	41.9	33.8
Completely important	28.0	48.8	36.7

*p*-value corresponds to the (bilateral) asymptotic significance obtained in Pearson’s chi-squared test.

## Supplementary Materials

The following are available online at www.mdpi.com/1660-4601/14/11/1313/s1, Table S1: Frequency of consumption of the foods considered by the AHEI with statistical differences among mothers in all three clusters, Table S2, Frequency of consumption of the foods considered by the AHEI with statistical differences among fathers in all three clusters, Table S3: Frequency of consumption of the foods considered by the AHEI with statistical differences among children in all three clusters.

## Appendix A.

**QUESTIONNAIRE**
**FOOD HABITS AND FAMIY SATISFACTION**
**FONDO DE DESARROLLO CIENTIFICO Y TECNOLOGICO (FONDECYT) 1160005**

**How old are you?**

_______ years

**State how often you eat the following foods:**

	**Daily Consumption**	**Three or More Times per Week, but Not Daily**	**Once or Twice per Week**	**Less than Once per Week**	**Never or Almost Never**
1. Cereals and derivatives					
2. Vegetables					
3. Fruit					
4. Milk and dairy products					
5. Meats					
6. Legumes					
7. Sausages and cold meats					
8. Sweets					
9. Soft drinks with sugar					

**Indicate your degree of agreement or disagreement with the following statements regarding your life** (one answer per row):

	**Disagree**			**Agree**		
	**Completely**	**Mostly**	**Slightly**	**Slightly**	**Mostly**	**Completely**
1. In most ways my life is close to my ideal						
2. The conditions of my life are excellent						
3. I am satisfied with my life						
4. So far I have gotten the important things I want in life						
5. If I could live my life over, I would change almost nothing						

**Indicate your degree of agreement or disagreement with the following statements regarding your diet** (one answer per row):

	**Disagree**			**Agree**		
	**Completely**	**Mostly**	**Slightly**	**Slightly**	**Mostly**	**Completely**
1. Food and meals are very positive elements in my life						
2. I am generally pleased with my food						
3. My life in relation to food and meals is close to my ideal						
4. Regarding food, the conditions of my life are excellent						
5. Food and meals bring great satisfaction to my daily life						

**Indicate your degree of agreement or disagreement with the following statements regarding your diet** (one answer per row):

	**Disagree**			**Agree**		
	**Completely**	**Mostly**	**Slightly**	**Slightly**	**Mostly**	**Completely**
1. In most ways my family life is close to my ideal						
2. The conditions of my family life are excellent						
3. I am satisfied with my family life						
4. So far I have gotten the important things I want in family life						
5. If I could live my family life over, I would change almost nothing						

**In general terms**:

	**Importance**					
	**Not Important**	**Very Little**	**Some**	**Moderate**	**Very**	**Extremely Important**
How important is food to your well-being?						

**According to your knowledge, health experts recommend consuming …**

	**More**	**Almost the Same**	**Less**	**Try to Avoid**	**Not Sure**
1. Fat					
2. Polyunsaturated fats					
3. Calories					
4. Sodium					
5. Saturated fat					
6. Whole grains					
7. Salt					
8. Trans fat					
9. Sugar					
10. Omega-3 fatty acids					
11. Fiber					
12. Monounsaturated fat					

**According to your knowledge, health experts recommend consuming …**

	**A lot**	**Some**	**Little**	**Try to Avoid**
1. Fruits and vegetables				
2. Starchy foods such as bread, rice, pasta, potatoes				
3. Protein sources				
4. Milk and dairy products				
5. Foods and drinks high in fat				
6. Foods and drinks high in sugar				
7. Foods and drinks high in salt				

**The following questions are related to the frequency of meals shared with family, and what you eat. In a typical week (seven days), how many days do you...?**

	**0 Day**	**1 Day**	**2 Days**	**3 Days**	**4 days**	**5 Days**	**6 Days**	**7 Days**
1. Eat home-made food								
2. Buy ready-to-eat food (i.e., from supermarkets)								
3. Order food at home (i.e., pizza or sandwiches)								
4. Eat in the home of friends or family								
5. Eat at restaurants								
6. Eat at fast-food outlets								

**How many times in the last seven days has your entire family gathered to eat?**

	**0 Day**	**1 Day**	**2 Days**	**3 Days**	**4 Days**	**5 Days**	**6 Days**	**7 Days**
1. Breakfast								
2. Lunch								
3. Dinner								

**If in the previous question you answered fewer than seven days, where do you usually eat these meals during the week?** (Mark only one answer per column).

	**Breakfast**	**Lunch**	**Dinner**
1. In my workplace (cafeteria or similar)			
2. In a restaurant near my workplace			
3. Buy food from a street vendor near my workplace			
4. In a fast food outlet near my workplace			
5. Buy sweets in a store near my workplace			
6. Bring food or snacks from home to eat at work			
7. I skip this meal			
8. Other (specify)			
If you answered “other” please indicate where			

**How many people are in your household (including yourself)? ________**

**How many children do you have? ________**

**Who decides what food to buy for your home? _________________________**

**Who purchases the food for your home? _________________________**

**Approximately how much do you spend on food for your home per month?**

**$_____________**

**Indicate your approximate weight and height:**

Weight________kg  Height_____m

**What is the work of the person who provides the main income for your household?**

	Only occasional or informal work
	Unqualified worker, minor trades, day laborer
	Qualified worker, junior foreman, micro-entrepreneur
	Low-level administrative employee, clerk, secretary, salesperson
	Mid-level executive, management, traditional professional
	Senior executive, general manager
	Currently looking for work
	Other situation (specify)_____________________________

**What is the education level of the person who provides the main income for your household?**

	Basic education incomplete or inferior
	Basic education complete
	High school incomplete
	High school complete or technical degree incomplete
	University incomplete or technical degree complete
	University complete
	Post-graduate (Masters, Doctorate or equivalent)
	Basic education incomplete or inferior
	Basic education complete
	High school incomplete
	High school complete or technical degree incomplete
	University incomplete or technical degree complete
	University complete
	Post-grad (Masters, Doctorate or equivalent)

**Considering the ancestry of your parents and grandparents, what is your origin...?**

	Mapuche
	Chilean
	Spanish
	German
	Italian
	Other (specify):

**THANK YOU FOR YOUR COLLABORATION**
