# Supplementary material for: Diet Quality and Satisfaction with Life, Family Life, and Food-Related Life across Families: A Cross-Sectional Pilot Study with Mother-Father-Adolescent Triads
Source: Int J Environ Res Public Health. 2017 Oct 29;14(11):1313. doi: 10.3390/ijerph14111313 (PMC5707952; doi:10.3390/ijerph14111313)
Supplement: Supplementary file 1 [file ijerph-14-01313-s001.pdf]

Table S1: Frequency of consumption of the foods considered by the AHEI with statistical differences among mothers in all three clusters.

Food	Group 1 (n = 118)	Group 2 (n = 43)	Group 3 (n = 139)
Cereals and derivatives		*P = 0.000*	
Daily consumption	7.6	46.5	23.0
Three or more times a week, but not daily	22.0	23.3	19.4
Once or twice a week	28.0	23.3	35.3
Less than once a week	19.5	2.3	5.8
Never or almost never	22.9	4.7	16.5
Vegetables		*P = 0.000*	
Daily consumption	39.8	88.4	65.5
Three or more times a week, but not daily	40.7	11.6	24.5
Once or twice a week	16.1	0.0	9.4
Less than once a week	34	0.0	0.7
Fruit		*P = 0.000*	
Daily consumption	28.0	76.7	50.4
Three or more times a week, but not daily	35.6	16.3	30.2
Once or twice a week	22.9	4.7	15.1
Less than once a week	11.9	2.3	2.2
Never or almost never	1.7	0.0	2.2
Milk and dairy products		*P = 0.000*	
Daily consumption	25.4	74.4	30.9
Three or more times a week, but not daily	27.1	11.6	33.8
Once or twice a week	28.8	14.0	18.7
Less than once a week	11.9	0.0	8.6
Never or almost never	6.8	0.0	1.0
Meats		*P = 0.012*	
Daily consumption	22.0	9.3	9.4
Three or more times a week, but not daily	44.9	41.9	42.4
Once or twice a week	22.9	46.5	34.5
Less than once a week	7.6	2.3	12.2
Never or almost never	2.5	0.0	1.4
Sausages and cold meats		*P = 0.000*	
Daily consumption	22.0	7.0	6.5
Three or more times a week, but not daily	25.4	7.0	23.0
Once or twice a week	23.7	20.9	23.0
Less than once a week	13.6	37.2	25.9
Never or almost never	15.3	27.9	21.6
Sweets		*P = 0.000*	
Daily consumption	11.0	0.0	6.5
Three or more times a week, but not daily	24.6	16.3	15.1
Once or twice a week	29.7	7.0	27.3

Less than once a week	15.3	41.9	22.3
Never or almost never	19.5	34.9	28.8
Soft drinks with sugar		*P = 0.000*	
Daily consumption	39.8	2.3	13.7
Three or more times a week, but not daily	16.9	2.3	15.1
Once or twice a week	11.9	14.0	25.9
Less than once a week	12.7	20.9	18.7
Never or almost never	18.6	60.5	26.6

Table S2: Frequency of consumption of the foods considered by the AHEI with statistical differences among fathers in all three clusters.

Food	Group 1 (n = 118)	Group 2 (n = 43)	Group 3 (n = 139)
Cereals and derivatives		*P = 0.001*	
Daily consumption	7.6	34.9	17.3
Three or more times a week, but not daily	23.7	27.9	15.1
Once or twice a week	21.2	18.6	23.7
Less than once a week	17.8	7.0	12.2
Never or almost never	29.7	11.6	31.7
Vegetables		*P = 0.012*	
Daily consumption	38.1	69.8	53.2
Three or more times a week, but not daily	36.4	27.9	28.1
Once or twice a week	19.5	2.3	16.5
Less than once a week	5.1	0.0	2.2
Never or almost never	0.8	0.0	0.0
Fruit		*P = 0.000*	
Daily consumption	17.8	51.2	26.8
Three or more times a week, but not daily	33.9	27.9	44.9
Once or twice a week	30.5	16.3	16.7
Less than once a week	13.6	4.7	8.0
Never or almost never	4.2	0.0	3.6
Milk and dairy products		*P = 0.018*	
Daily consumption	16.9	37.2	34.5
Three or more times a week, but not daily	21.2	27.9	22.3
Once or twice a week	33.1	18.6	20.9
Less than once a week	17.8	14.0	11.5
Never or almost never	11.0	2.3	10.8
Sausages and cold meats		*P = 0.005*	
Daily consumption	28.0	11.6	13.7
Three or more times a week, but not daily	21.2	11.6	29.5
Once or twice a week	26.3	23.3	23.0
Less than once a week	15.3	34.9	23.7
Never or almost never	9.3	18.6	10.1

Soft drinks with sugar		*P = 0.000*	
Daily consumption	45.8	4.7	22.3
Three or more times a week, but not daily	20.3	14.0	23.0
Once or twice a week	16.9	9.3	15.8
Less than once a week	8.5	18.6	21.6
Never or almost never	8.5	53.5	17.3

P value corresponds to the (bilateral) asymptotic significance obtained in Pearson's Chi squared Test

Table S3: Frequency of consumption of the foods considered by the AHEI with statistical differences among children in all three clusters.

Food	Group 1 (n = 118)	Group 2 (n = 43)	Group 3 (n = 139)
Cereals and derivatives		*P = 0.000*	
Daily consumption	13.6	58.1	33.8
Three or more times a week, but not daily	22.0	30.2	29.5
Once or twice a week	33.9	4.7	29.5
Less than once a week	10.2	0.2	5.8
Never or almost never	20.3	7.0	7.2
Vegetables		*P = 0.000*	
Daily consumption	22.0	76.7	50.4
Three or more times a week, but not daily	37.3	18.0	34.5
Once or twice a week	26.3	4.0	13.7
Less than once a week	5.9	0.7	0.7
Never or almost never	8.5	0.6	0.7
Fruit		*P = 0.000*	
Daily consumption	13.6	81.4	43.2
Three or more times a week, but not daily	24.6	11.6	36.8
Once or twice a week	42.4	4.7	14.4
Less than once a week	15.3	2.0	2.9
Never or almost never	4.2	0.3	0.7
Milk and dairy products		*P = 0.000*	
Daily consumption	29.7	83.7	74.8
Three or more times a week, but not daily	24.6	11.6	14.4
Once or twice a week	22.9	2.3	8.0
Less than once a week	12.7	2.0	2.2
Never or almost never	4.4	0.3	0.6
Sausages and cold meats		*P = 0.000*	
Daily consumption	16.1	4.7	6.5
Three or more times a week, but not daily	31.4	11.6	29.5
Once or twice a week	23.7	18.6	33.1
Less than once a week	19.5	25.6	16.5
Never or almost never	9.3	39.5	14.4

Sweets		*P = 0.000*	
Daily consumption	26.3	4.7	15.8
Three or more times a week, but not daily	28.8	16.3	25.9
Once or twice a week	28.0	9.3	26.5
Less than once a week	11.0	32.6	19.4
Never or almost never	5.9	37.2	12.2
Soft drinks with sugar		*P = 0.000*	
Daily consumption	47.5	7.0	23.0
Three or more times a week, but not daily	22.9	2.3	24.5
Once or twice a week	16.9	20.9	21.6
Less than once a week	9.3	20.9	17.3
Never or almost never	3.4	48.8	13.7

P value corresponds to the (bilateral) asymptotic significance obtained in Pearson's Chi squared Test
